# Editorial: Improving stem cell transplantation delivery using computational modelling

**DOI:** 10.3389/fimmu.2025.1579353

**Published:** 2025-03-10

**Authors:** Pawan Kumar Raghav

**Affiliations:** Immunogenetics and Transplantation Laboratory, Department of Surgery, University of California San Francisco, San Francisco, CA, United States

**Keywords:** Hematopoietic stem cells (HSCs), HSC transplantation (HSCT), regenerative medicine, cancer stem cells (CSCs), immunomodulation, graft-versus-host disease (GVHD), computational modeling, machine learning

Stem cell transplantation has emerged as a cornerstone of regenerative medicine due to its ability to differentiate into various cell types and its potential applications in immune modulation, treating immunological disorders, and hematological malignancies (1). Among various stem cell types, pluripotent embryonic stem cells (ESCs) and multipotent adult stem cells (ASCs) have been extensively studied for their differentiation potential. ESCs possess superior pluripotency, allowing them to generate any cell type in the human body. However, ethical concerns surrounding their use have led to a greater focus on alternative sources such as induced pluripotent stem cells (iPSCs) and ASCs, including mesenchymal stem cells (MSCs), neural stem cells (NSCs), and hematopoietic stem cells (HSCs). MSCs have shown immunomodulatory effects by modulating T, B, natural killer (NK), and dendritic cells, making them a promising tool for autoimmune and inflammatory disorders (2, 3). HSCs from human umbilical cord blood have been widely used in transplantation therapies for hematopoietic and immune-related diseases (4). The success of HSC transplantation (HSCT) hinges on homing, migration, engraftment, self-renewal, and differentiation. These complex processes are regulated by growth factors, cytokines, and niche interactions. Despite HSCT’s therapeutic potential, challenges like graft-versus-host disease (GVHD), graft rejection, and variable patient outcomes persist. Strategies such as immune tolerance induction and genetic, and therapeutic modifications are being explored to enhance stem cell survival and integration (5–8). Recent advancements suggest that integrating computational models with immunological data has opened new avenues to improve stem cell engraftment (9). Machine learning models enable the identification of key transcription factors and gene networks involved in self-renewal and lineage specification in regenerative medicine (10, 11). These approaches also facilitate the comparison of healthy stem cells and cancer stem cells (CSCs), aiding in the development of targeted therapies for malignancies (12, 13).

The Frontiers in Immunology Research Topic, “*Improving Stem Cell Transplantation Delivery Using Computational Modelling*” exemplifies this interdisciplinary approach and brings together pioneering studies in a series of compiled articles, contributing unique insights into the field. A notable study by Stiehl et al. reported mechanistic computational simulation models that explore how variations in stem cell graft characteristics influence clonal dynamics post-allogeneic HSCT (allo-HSCT). This study suggests that fewer transplanted HSCs per kilogram of recipient body weight may increase donor-derived clones’ expansion. Conversely, the dose of transplanted progenitor cells and post-transplantation cytokine support appear to have minimal impact on clonal dynamics. Additionally, the study indicates a correlation between changes in donor variant allele frequencies prior to donation and clonal expansion in recipients. These findings underscore the importance of optimizing graft composition and dosing to improve transplantation outcomes. Rosenberger et al. conducted a comprehensive genome-wide association study (GWAS) to identify genetic factors influencing the success of HSCT. This uncovered several novel genomic loci significantly correlated with key transplant outcomes, including GVHD, overall survival rates, and graft rejection incidents. These findings highlight the genetic underpinnings affecting HSCT efficacy and pave the way for more personalized approaches in donor selection and pre-transplant conditioning regimens. Budeus et al. addressed the critical challenge of distinguishing MSCs from fibroblasts. Utilizing computational analysis, Budeus et al. identified unique gene expression signatures and signaling pathways that reliably differentiate between MSCs and fibroblasts. This differentiation is pivotal, as MSCs possess therapeutic potential due to their ability to home to injury sites and secrete regenerative factors, whereas fibroblasts lack these properties. The study’s findings enhance the precision of MSC identification, thereby improving the selection process for therapeutic applications and advancing the field of regenerative medicine. Chen et al. explored the application of artificial intelligence in identifying cancer stem cells (CSCs). Due to their self-renewal and differentiation capabilities, CSCs contribute to tumor initiation, relapse, and metastasis (14). This mini-review discussed various convolutional neural network (CNN)-based deep learning models developed to automate CSC recognition in biological images. This advancement enhances diagnostic precision and potentially informs targeted therapies, highlighting the transformative potential of combining computational modeling with immunological research in stem cell transplantation. Wang et al. conducted a retrospective analysis of consecutive myelodysplastic syndrome (MDS) patients who underwent myeloablative allo-HSCT. The research evaluated the impact of different pre-transplant therapies and the timing of transplantation on patient outcomes. Findings revealed that patients receiving supportive care or hypomethylating agents (SC/HMA) with a transplantation interval of less than six months exhibited significantly improved overall survival (OS) and reduced non-relapse mortality (NRM) compared to other groups. These results suggest that implementing a pre-transplant strategy involving SC/HMA and proceeding to transplantation within six months of diagnosis may enhance survival rates and decrease transplant-related complications in MDS patients undergoing myeloablative allo-HSCT. Conclusively, future efforts should focus on personalized medicine that utilizes patient-specific data to create individualized models predicting responses to stem cell therapies. Additionally, the development of computational tools to monitor stem cell transplantation progress in real time will allow for timely interventions. The articles within this research topic exemplify the transformative potential of computational modeling in stem cell transplantation. By integrating advanced computational tools with experimental and clinical data, these studies pave the way for personalized and more effective therapeutic strategies, ultimately enhancing patient outcomes in stem cell transplantation.

## References

[1] Raghav PK Gangenahalli G. Hematopoietic stem cell molecular targets and factors essential for hematopoiesis. J Stem Cell Res Ther. (2018) 8:2. doi: 10.4172/2157-7633.1000441

[2] Raghav PK Mann Z Ahlawat S Mohanty S. Mesenchymal stem cell-based nanoparticles and scaffolds in regenerative medicine. Eur J Pharmacol. (2022) 918:174657. doi: 10.1016/j.ejphar.2021.174657 34871557

[3] Rawat S Jain KG Gupta D Raghav PK Chaudhuri R Pinky. Graphene nanofiber composites for enhanced neuronal differentiation of human mesenchymal stem cells. Nanomedicine. (2021) 16:1963–82. doi: 10.2217/nnm-2021-0121 34431318

[4] Mann Z Sengar M Verma YK Rajalingam R Raghav PK. Hematopoietic stem cell factors: their functional role in self-renewal and clinical aspects. Front Cell Dev Biol. (2022) 10:664261. doi: 10.3389/fcell.2022.664261 35399522 PMC8987924

[5] Wang P Jiang Z Wang C Liu X Li H Xu D. Immune tolerance induction using cell-based strategies in liver transplantation: clinical perspectives. Front Immunol. (2020) 11:1723. doi: 10.3389/fimmu.2020.01723 33013824 PMC7461870

[6] Raghav PK Singh AK Gangenahalli G. Stem cell factor and NSC87877 combine to enhance c-Kit mediated proliferation of human megakaryoblastic cells. PloS One. (2018) 13(11):e0206364. doi: 10.1371/journal.pone.0206364 30388134 PMC6214509

[7] Raghav PK Singh AK Gangenahalli G. Stem cell factor and NSC87877 synergism enhances c-Kit mediated proliferation of human erythroid cells. Life Sci. (2018) 214:84–97. doi: 10.1016/j.lfs.2018.09.055 30308182

[8] Raghav PK Gangenahalli G. PU. 1 mimic synthetic peptides selectively bind with GATA-1 and allow c-jun PU. 1 binding to enhance myelopoiesis. Int J Nanomed. (2021) 16:3833–59. doi: 10.2147/IJN.S303235 PMC8187006 34113102

[9] Garuffo L Leoni A Gatta R Bernardi S. The applications of machine learning in the management of patients undergoing stem cell transplantation: are we ready? Cancers. (2025) 17:395. doi: 10.3390/cancers17030395 39941764 PMC11816169

[10] Ouyang JF Chothani S Rackham OJ. Deep learning models will shape the future of stem cell research. Stem Cell Rep. (2023) 18:6–12. doi: 10.1016/j.stemcr.2022.11.007 PMC9860061 36630908

[11] Etzrodt M Endele M Schroeder T. Quantitative single-cell approaches to stem cell research. Cell Stem Cell. (2014) 15:546–58. doi: 10.1016/j.stem.2014.10.015 25517464

[12] Malta TM Sokolov A Gentles AJ Burzykowski T Poisson L Weinstein JN. Machine learning identifies stemness features associated with oncogenic dedifferentiation. Cell. (2018) 173:338–54. doi: 10.1016/j.cell.2018.03.034 PMC5902191 29625051

[13] Raghav PK Bhardwaj R Raghava GPS. Machine learning based identification of stem cell genes involved in stemness. J Cell Sci Ther. (2019) 3:25–6. doi: 10.4172/2157-7013-C1-049

[14] Raghav PK Mann Z. Cancer stem cells targets and combined therapies to prevent cancer recurrence. Life Sci. (2021) 277:119465. doi: 10.1016/j.lfs.2021.119465 33831426

